# The diagnostic and triage accuracy of digital and online symptom checker tools: a systematic review

**DOI:** 10.1038/s41746-022-00667-w

**Published:** 2022-08-17

**Authors:** William Wallace, Calvin Chan, Swathikan Chidambaram, Lydia Hanna, Fahad Mujtaba Iqbal, Amish Acharya, Pasha Normahani, Hutan Ashrafian, Sheraz R. Markar, Viknesh Sounderajah, Ara Darzi

**Affiliations:** 1 Department of Surgery & Cancer, Imperial College London, St. Mary’s Hospital, London, W2 1NY UK; 2 Institute of Global Health Innovation, Imperial College London, South Kensington Campus, London, SW7 2AZ UK; 3 Department of Molecular Medicine and Surgery, Karolinska Institutet, Stockholm, Sweden; 4 Nuffield Department of Surgery, Churchill Hospital, University of Oxford, OX3 7LE Oxford, UK

**Keywords:** Diagnosis, Health policy

## Abstract

Digital and online symptom checkers are an increasingly adopted class of health technologies that enable patients to input their symptoms and biodata to produce a set of likely diagnoses and associated triage advice. However, concerns regarding the accuracy and safety of these symptom checkers have been raised. This systematic review evaluates the accuracy of symptom checkers in providing diagnoses and appropriate triage advice. MEDLINE and Web of Science were searched for studies that used either real or simulated patients to evaluate online or digital symptom checkers. The primary outcomes were the diagnostic and triage accuracy of the symptom checkers. The QUADAS-2 tool was used to assess study quality. Of the 177 studies retrieved, 10 studies met the inclusion criteria. Researchers evaluated the accuracy of symptom checkers using a variety of medical conditions, including ophthalmological conditions, inflammatory arthritides and HIV. A total of 50% of the studies recruited real patients, while the remainder used simulated cases. The diagnostic accuracy of the primary diagnosis was low across included studies (range: 19–37.9%) and varied between individual symptom checkers, despite consistent symptom data input. Triage accuracy (range: 48.8–90.1%) was typically higher than diagnostic accuracy. Overall, the diagnostic and triage accuracy of symptom checkers are variable and of low accuracy. Given the increasing push towards adopting this class of technologies across numerous health systems, this study demonstrates that reliance upon symptom checkers could pose significant patient safety hazards. Large-scale primary studies, based upon real-world data, are warranted to demonstrate the adequate performance of these technologies in a manner that is non-inferior to current best practices. Moreover, an urgent assessment of how these systems are regulated and implemented is required.

## Introduction

Digital and online symptom checkers are application or software tools that enable patients to input their symptoms and biodata to produce a set of differential diagnoses and clinical triage advice. The diagnostic function of symptom checkers is to provide a list of differential diagnoses, ranked by likelihood^1^. The triage function highlights to end-users the most appropriate course of action regarding their potential diagnosis, which typically includes seeking urgent care; contacting their general practitioner; or self-care. Symptom checkers have become an increasingly prominent feature of the modern healthcare landscape due to increasing access to internet connectivity and a cultural shift towards more involved self-care engagement^2^. In 2020, 96% of UK households had internet access, of which over one-third of adults used the internet to self-diagnose health-related issues^2,3^. Governments have also incorporated symptom checkers into their formal health and social care pathways in order to alleviate the increasing burden that is placed upon both primary care services and emergency services, particularly in light of the COVID-19 pandemic^4–6^. In 2017, the NHS 111 triage service, backed by Babylon, reported over 15,500 app downloads and was responsible for over 9700 triages^7^. In 2021, Babylon reportedly covered 4.3 million people worldwide, performing over 1.2 million digital consultations with 4000 clinical consultations each day, with one patient interaction every 10 s.

It has been previously estimated that 12% of emergency department (ED) attendances would be more appropriately managed at other sites of care^8,9^. Hence, symptom checkers have the potential to reduce the financial and resource burden placed upon hospitals and can help focus resources towards those who are truly in medical need. Public and private companies have advertised symptom checkers to be a cost-effective solution that may serve as a first port-of-call for patients and effectively signpost patients to the most appropriate healthcare service. When used appropriately, symptom checkers can advise patients with serious conditions to seek urgent attention and conversely prevent those with problems best resolved through self-care from unnecessarily seeking medical attention^1^.

However, for all of the potential health, organisational and financial benefits, symptom checkers are heavily dependent on the accuracy of diagnostic and triage advice that they provide. Overtriaging those with non-urgent ailments will exacerbate the unnecessary use of healthcare services. Conversely and more seriously, inaccuracies in diagnosing and triaging patients with life-threatening conditions could result in preventable morbidity and mortality^1,10^. In fact, symptom checkers have previously received heavy media criticism for not correctly diagnosing cancer, cardiac conditions, and providing differing advice to patients with the same symptomatology but different demographic characteristics^11–13^. These alleged reports raise concerns around the possibility that these systems may deliver unequitable clinical performance across different gender and sociodemographic groups. In a previous systematic review, Chambers et al. (2019) assessed symptom checkers on their safety and ability to correctly diagnose and distinguish between high and low acuity conditions^14^. The diagnostic accuracy was found to be variable between different platforms and was generally low. Given the rapid expansion in commercially available digital and online symptom checkers, a more updated review is warranted to determine if this is still the case. Thus, this review aims to systematically evaluate the currently available literature regarding (1) the accuracy of digital and online symptom checkers in providing diagnoses and appropriate triage advice as well as (2) the variation in diagnoses and triage recommendations provided by differing symptom checkers given identical clinical input data within the same study.

## Results

### Search

**Fig. 1:**
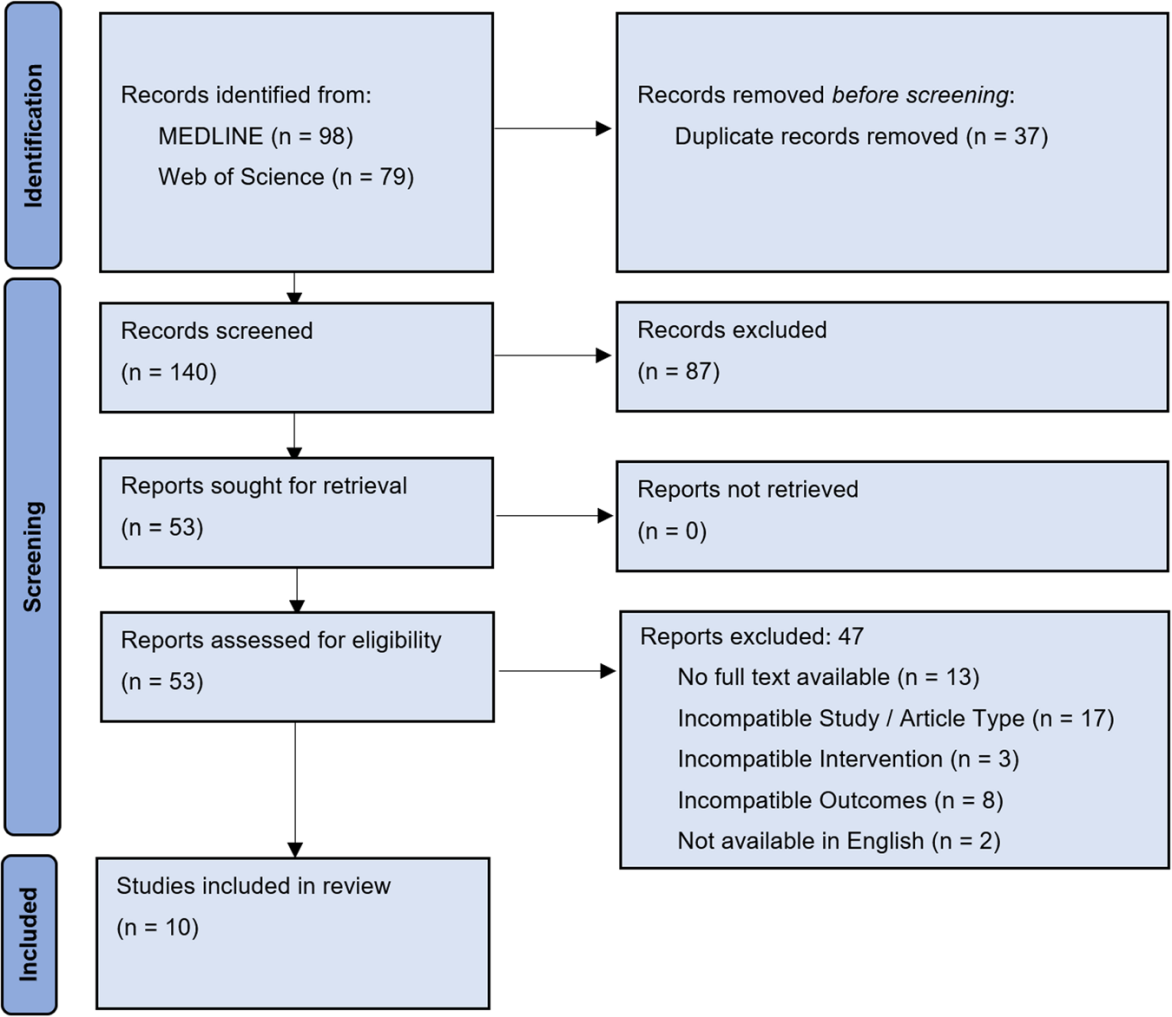
Preferred reporting items for Systematic Reviews and Meta-Analysis (PRISMA) flow diagram showing the process of study selection for this systematic review of symptom checker diagnostic and triage accuracy.

**Fig. 2:**
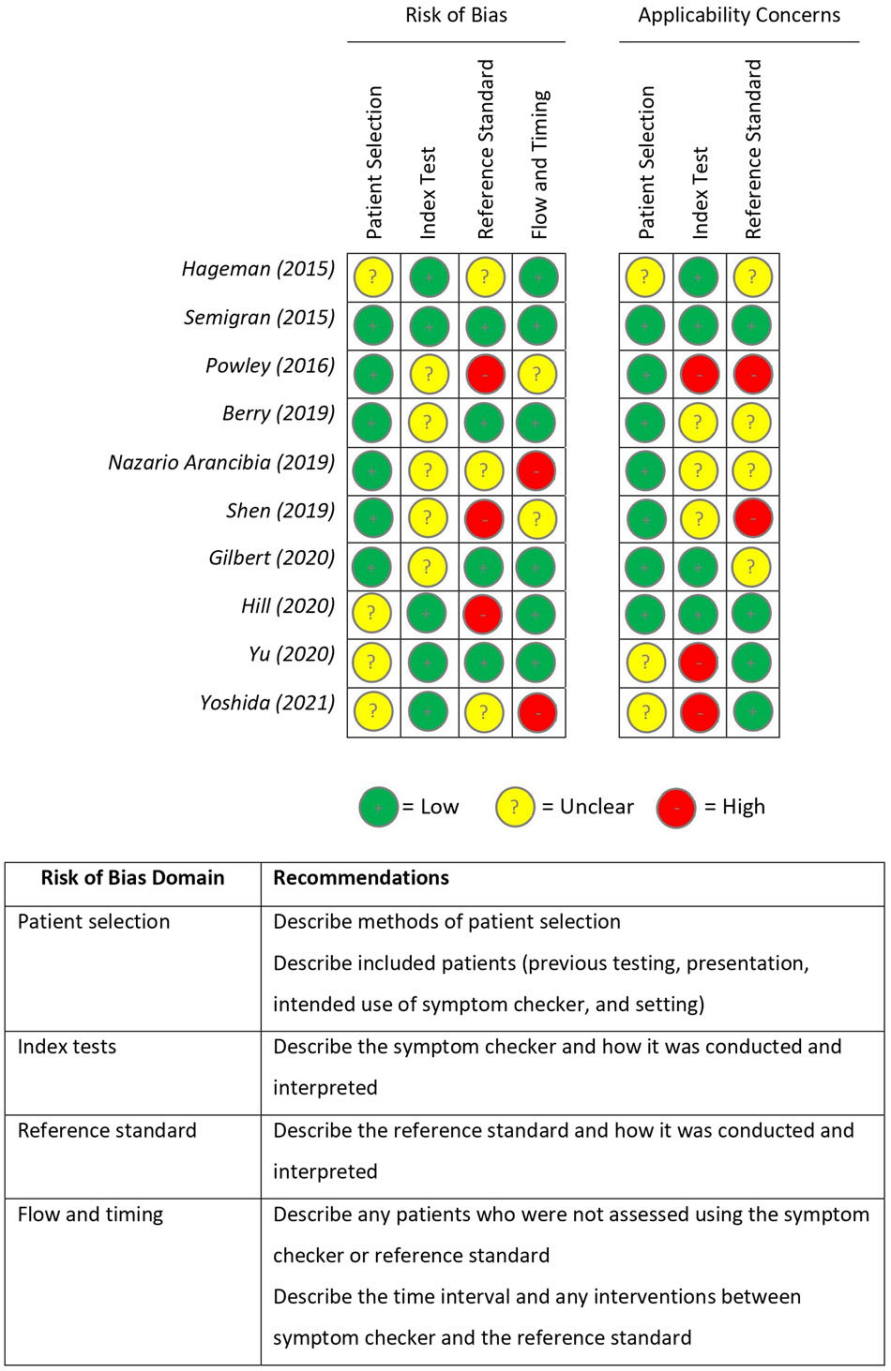
Risk of bias summary using QUADAS-2 risk assessment tool^44^. Authors’ judgement regarding each domain of bias of each study synthesised on the accuracy of symptom checkers. Risk was categorised into one of three categories: low risk (+), unclear risk (?) and high risk (−). The table shows possible items to consider in future work involving symptom checkers to achieve a low risk of bias or applicability concerns.

The literature search yielded ten studies that met the inclusion criteria. Figure 1 presents the flow of studies through the screening process. An overview of the risk of bias assessment using QUADAS-2 can be found in Fig. 2. Most studies had domains of ‘unclear’ or ‘high’ risk of bias or applicability concerns. Six studies had one or more domains at a ‘high’ risk of bias^15–20^.

### Characteristics of participants and interventions

Characteristics of included studies can be found in Table 1. Study population size ranged from 27 to 214 patients or vignettes^16,20^. Three studies were conducted by researchers in USA^1,21,22^, and one each in Australia^18^, Canada^17^, Hong Kong^19^, Spain^16^, and the UK^15^. The remaining two were multinational studies^20,23^.

**Table 1:** Characteristics of the ten studies included in the systematic review on the diagnostic and triage accuracy of symptom checkers.

First author (year)	Study design	Participants	Intervention	Symptom checker(s) used	Comparator	Outcome measures
Hageman (2015)^21^	Prospective cross-sectional study	86 hand clinic patients	Outpatients prospectively input data and symptoms into symptom checker to guess diagnosis	WebMD	Diagnosis from a hand surgeon	Diagnostic accuracy (top 3)
Semigran (2015)^1^	Vignette cross-sectional study	45 standardised vignettes: 15 emergency 15 non-emergency 15 self-care	Patient data and symptoms from various cases input into 23 symptom checkers	Ask MD, BetterMedicine. DocResponse, Doctor Diagnose, Drugs.com, EarlyDoc, Esagil, Family Doctor, FreeMD, HMS Family Health Guide, Healthline, Healthwise, Healthy Children, Isabel, iTriage, Mayo Clinic, MEDoctor, NHS Symptom Checkers, Steps2Care, Symcat, Symptify, Symptomate, WebMD	True diagnosis and appropriate triage advice	Diagnostic accuracy (primary diagnosis, top 3 and top 20 results) Triage accuracy (overall, emergency, non-emergency, self-care)
Powley (2016)^15^	Prospective cross-sectional study	34 inflammatory arthritis patients	Patients completed NHS and WebMD symptom checkers with their presenting symptoms	NHS Choices, WebMD	Diagnosis from secondary care	Diagnostic accuracy (primary diagnosis, top 5)
Berry (2019)^22^	Retrospective cross-sectional study	ED records of 168 HIV/hepatitis C patients	Retrospective input of patient data into symptom checkers	Mayo Clinic, WebMD, Symptomate, Symcat, Isabel	Emergency department physician determined diagnosis	Diagnostic accuracy (primary diagnosis, top 3 and top 10) Triage accuracy
Nazario Arancibia (2019)^16^	Prospective cross-sectional study	214 low-priority ED patients	Patients interviewed using questions from symptom checker to produce differentials	Mediktor	Emergency department diagnosis	Diagnostic accuracy (primary diagnosis, top 3, top 5, and top 10)
Shen (2019)^17^	Vignette cross-sectional study	42 vignettes of ophthalmic conditions	Cases inputted into symptom checker to record results	WebMD	True diagnosis and appropriate triage advice	Diagnostic accuracy (primary diagnosis and top 3). Triage accuracy (emergency, non-emergency)
Gilbert (2020)^23^	Vignette cross-sectional study	200 primary care vignettes	Vignettes used to input into symptom checkers and to assess general practitioners (GPs)	ADA, Babylon, Buoy, K health, Mediktor, Symptomate, WebMD, YourMD	True diagnosis and GPs	Diagnostic accuracy (primary diagnosis, top 3 and top 5) Triage accuracy
Hill (2020)^18^	Vignette cross-sectional study	48 vignettes with ‘Australia-specific conditions’	Vignettes summarised and entered in symptom checkers	36 symptom checkers, 17 diagnostic only, 9 triage only advice. 10 that provide both.	True diagnosis and appropriate triage advice	Diagnostic accuracy (primary diagnosis, top 3 and top 10) Triage accuracy (emergency, non-emergency, self-care)
Yu (2020)^19^	Retrospective cross-sectional study	100 ED patient records	Retrospectively using patient records to input information into symptom checkers	Drugs.com FamilyDoctor	Given ED triage level	Triage accuracy
Yoshida (2021)^20^	Vignette cross-sectional study	27 vignettes of orofacial pain conditions	Vignettes of varied orofacial conditions input into symptom checkers	Esagil, FreeMD, Healthline, Isabel, Mayo Clinic, MEDoctor, Symcat, Symptify, Symptomate, WebMD, ADA	True diagnosis by resident in orofacial pain and oral medicine	Diagnostic accuracy (primary diagnosis, top 4)

Three studies involved prospective data collection (i.e., from outpatient clinics and ED waiting areas)^15,16,21^, two studies involved retrospective analysis of ED clinical assessments^19,22^, and five studies used ‘simulated’ patient vignettes (i.e., cases written by clinicians)^1,17,18,20,23^. The pathologies evaluated included hand conditions^21^, inflammatory arthritides^15^, infectious diseases (HIV and Hepatitis C)^22^, ophthalmic conditions^17^, and orofacial conditions^20^. Four studies examined a wide range of general medical conditions pertinent to ED and general practice^1,18,19,23^.

A total of 48 different symptom checkers were utilised in the included studies. Three studies only used one symptom checker^16,17,21^, while two studies used more than 20 symptom checkers^1,18^. Of note, the WebMD symptom checker was assessed eight times and was the most commonly assessed symptom checker in this review^1,15,17,18,20–23^.

### Diagnostic accuracy

**Fig. 3:**
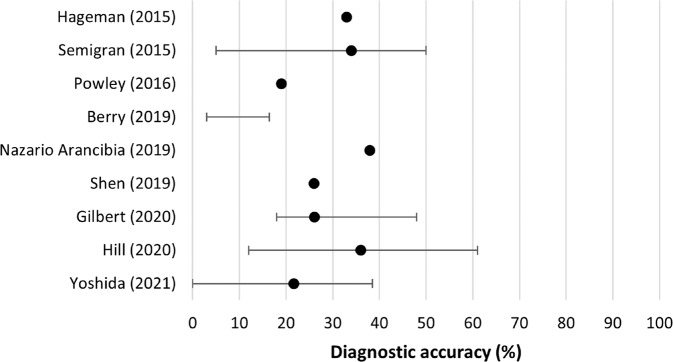
Mean primary diagnostic accuracy of symptom checkers in each study. Error bars signify the range of accuracy of different symptom checkers for the same patient/vignette population. An overall accuracy value was not given in Berry (2019).

Nine studies evaluated symptom checker diagnostic accuracy (Table 2). Overall primary diagnostic accuracy (i.e., listing the correct diagnosis first) was low in all studies, ranging from 19 to 38% (Fig. 3)^15,16^. Top three diagnostic accuracy, measured in seven studies, ranged from 33 to 58%^16,21^. Diagnostic accuracy for each specific symptom checker can be found in Supplementary Table 1.

**Table 2:** Overall and range of average diagnostic and triage accuracy of symptom checkers in each study.

First author (year)	No. of symptom checkers	Overall average diagnostic accuracy (%)	Range of average diagnostic accuracy (%)	Average triage accuracy (%)	Range of average triage accuracy (%)
Hageman (2015)^21^	1	33	n/a	ns	n/a
Semigran (2015)^1^	23	34	5–50	57	33–78
Powley (2016)^15^	1	19	n/a	ns	n/a
Berry (2019)^22^	5	ns	3–16.4	48.8	ns
Nazario Arancibia (2019)^16^	1	37.9	n/a	ns	n/a
Shen (2019)^17^	1	26	n/a	66.7	n/a
Gilbert (2020)^23^	8	26.1	18–48	90.1	80–97.8
Hill (2020)^18^	36	36	12–61	49	17–61
Yu (2020)^19^	2	ns	ns	62	50–74
Yoshida (2021)^20^	11	21.7	0–38.5	ns	ns

*n/a* not applicable as only one symptom checker was used, *ns* not stated.

### Triage accuracy

**Fig. 4:**
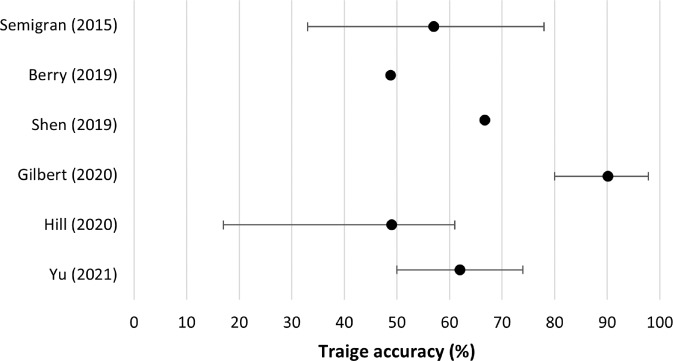
Mean accuracy of triage information given by symptom checkers in each study. Error bars signify the range of accuracy of different symptom checkers for the same patient/vignette population.

Six studies examined the accuracy of symptom checkers in providing correct triage advice (Table 2). Overall triage accuracy tended to be higher than diagnostic accuracy, ranging from 49 to 90% (Fig. 4)^18,22,23^. Three studies stratified findings by emergency and non-emergency cases. Two studies reported that emergency cases were triaged more accurately (mean percentage [95% CI]) than non-urgent cases (80% [75–86] versus 55% [47–63], and 63% [52–71] versus 30% [11–39], respectively)^1,18^. However, another study demonstrated that the accuracy of triage advice for ophthalmic emergencies was significantly lower than in non-urgent conditions (39% [14–64] versus 88% [73–100])^17^. Triage accuracy for each specific symptom checker can be found in Supplementary Table 2.

### Variation in accuracy

All five studies that reported >1 symptom checker demonstrated marked variability in diagnostic accuracy among different symptom checkers for the same patient vignettes^1,18,20,22,23^. For a standardised set of general medical vignettes, mean primary diagnostic accuracy ranged from 5 to 50%^1^. Accuracy for diagnosing primary care conditions ranged from 18 to 48%^23^. Correct diagnosis of infectious diseases ranged from 3 to 16%^22^. Finally, primary diagnostic accuracy of different symptom checkers assessing orofacial conditions ranged from 0 to 38.5%^20^. Similarly, the accuracy of triage advice given by individual symptom checkers varied within a study. Semigran et al. and Hill et al. found a range of 33 to 78% and 17 to 61%, respectively, for general medical condtions^1,18^. Gilbert et al. reported a mean triage accuracy range of 80–97% for 200 primary care vignettes^23^. Variations in accuracy of providing diagnoses and triage advice were also apparent for specific symptom checkers examining different conditions. The WebMD symptom checker was most frequently used and was assessed in eight studies. The primary diagnostic accuracy of WebMD ranged from 3 to 53% across a variety of medical conditions that were assessed in eight included studies (Table 3)^1,22^.

**Table 3:** Primary diagnostic accuracy of WebMD in included studies.

First author (year)	Participants	WebMD primary diagnostic accuracy (%)
Hageman (2015)^21^	86 hand clinic patients	33
Semigran (2015)^1^	45 standardised vignettes:	36
	15 emergency	
	15 non-emergency	
	15 self-care	
Powley (2016)^15^	34 inflammatory arthritis patients	19
Berry (2019)^22^	ED records of 168 HIV/hepatitis C patients	3 (Hep C), 7.8 (HIV), 7.1 (both)
Shen (2019)^17^	42 vignettes of ophthalmic conditions	26
Gilbert (2020)^23^	200 primary care vignettes	21
Hill (2020)^18^	48 vignettes with ‘Australia-specific conditions’	53
Yoshida (2021)^20^	27 vignettes of orofacial pain conditions	30.77

## Discussion

This systematic review evaluated the diagnostic and triage accuracy of symptom checkers for a variety of medical conditions using both simulated and real-life patient vignettes. Our review highlighted that both diagnostic and triage accuracies were generally low. Moreover, there is considerable variation across symptom checkers despite being presented with uniform symptom parameters. We also note that the diagnostic and triage accuracies of symptom checkers, as well as the variation in performance, were greatly dependent on the acuity of the condition assessed. As a whole, these issues raise multiple concerns about the use of symptom checkers as patient-facing tools, especially given their increasingly endorsed role within health systems as triage services that direct patients towards appropriate treatment pathways.

It can be argued that the accuracy of triage advice given by symptom checkers is of greater importance compared to diagnostic accuracy, especially considering the increasing role of symptom checkers as triage services that direct users towards appropriate treatment. This is significant for acute and life-threatening conditions, where the ability of a symptom checker to classify between the need to seek urgent medical attention versus non-urgent medical attention is particularly important. Moreover, as attaining an accurate diagnosis of certain pathologies (e.g., HIV, autoimmune conditions, malignancies) by clinicians would typically involve further biochemical or radiological investigation; it would be arguably unrealistic to expect symptom checkers to be accurate in their diagnosis of the full spectrum of medical conditions. Thus, the onus on symptom checkers should be placed on triage advice accuracy, rather than accurate diagnosis, to ensure users are signposted towards appropriate healthcare pathways.

However, when unsafe triage advice is paired with an incorrect set of differential diagnoses, this alignment of errors increases the likelihood of clinical harm to patients, not unlike the Swiss-Cheese model that is cited in aviation safety reports^24^. For example, in the UK, Babylon, an NHS-backed symptom checker, has been alleged to suggest that a breast lump may not necessarily represent cancer and it has also been reported to have misinterpreted myocardial infarctions as panic attacks^11,12^. While there will be instances where probability-based clinical decision-making tools are incorrect, a safety-first approach needs to be employed for specific high-risk conditions, particularly when processing low-risk symptoms that may mask or mimic more life-threatening problems.

Variability in accuracy is a concerning recurrent theme in the included studies and indicates that patients are provided with heterogeneous advice, dependent on the symptom checker used and the condition assessed, which results in a spectrum of issues. There is a caveat that such variability is demonstrated through values that are based on different vignettes, time-points, and research teams, all of which introduce confounding factors that require accounting for in future work. However, despite this, variability combined with poor diagnostic and triage accuracy presents a multidimensional system of potential patient harm. Although ‘undertriaging’ has clearly appreciable deleterious effects on patient wellbeing, it is worth noting that ‘overtriaging’ manifests in inappropriate health resource utilisation through unnecessary presentation to emergency services. Even if this does not impact the health of the primary symptom checker user, it does confer a knock-on opportunity cost that is shouldered by those truly in need of emergency services and are left waiting for medical attention. Although the impact of variable triaging advice from symptom checkers has yet to be robustly researched, the highly varied accuracy between symptom checkers noted in this review suggests that there is considerable scope for discrepancies in quality and health outcomes.

Many cite the poor transparency and reporting of both symptom checker development and subsequent clinical validation as issues that severely limit the extent to which these technologies can be meaningfully endorsed for population-wide use within health systems. Minimal evidence is provided regarding the context, patient demographics and clinical information that is used to create symptom checkers. Case coverage (i.e., what conditions and patient populations are accounted for by the software) must be explained, especially since symptom checkers may not account for geographical or country-specific variations in disease prevalence, thus impacting generalisability and potential utility. Moreover, symptom checkers are not explainable in how they arrive to their recommendations. A focus on explainability would significantly increase the ability to effectively audit these devices as well as increase trust from both patients and healthcare professionals in the outputs they provide. Overall trust is also hindered by claims of several symptom checkers to purported house ‘AI algorithms’ as part of their diagnostic process, despite not providing any convincing evidence for this, again reflecting the poor transparency and reporting of the symptom checker development and validation process.

In their current guise, symptom checkers are at best considered as an adjunctive technology which may direct patients towards various services with an appropriate level of urgency and reduce the burden placed on EDs and primary care services. This is especially relevant in areas where access to traditional assessment models may be scarce. However, our study has highlighted several safety and regulatory concerns in their potential use, particularly around variation between different symptom checkers and the conditions being assessed.

### Study limitations

First, five of the included studies used simulated patient vignettes, which are unlikely to represent the complexity of real patients^25–27^. Future work could include real-life patient data to increase software exposure to the nuances of real-life patient populations. Second, bias is inevitable in retrospective studies, since much of the data being inputted rely on accurate documentation of patient details, their symptoms, and outcomes afterwards. Third, the observed variation between symptom checkers may be partly attributable to study heterogeneity, including the medical conditions studied, their context, and the way symptom details are inputted into the symptom checker (sequence, level of certainty and agreement). Fourth, several studies have suggested that diagnostic accuracy of symptom checkers may improve over time given increased exposure to data upon which an AI-centred model may iterate. This can lead to variance in performance that is hard to account for in a cross-sectional study. Fifth, this review did not capture all existing online symptom checkers. The internet is abundant with symptom checkers and although the majority do not have any validatory reports, newer and more improved symptom checkers to those studied may be available. Finally, our study synthesises data and findings from high-income countries given the paucity of data from middle and low-economically developed countries, which may exhibit different health-seeking behaviours, digital literacy rates, and disease burdens.

### Recommendations

#### For regulatory organisations

A reassessment of how symptom checkers are regulated is required so that the class of these devices better reflect the clinical function and associated risk that these systems serve in the real world as a public-facing source of medical advice^10,28^. Currently, global regulatory authorities, such as The Medicines and Healthcare Products Regulatory Agency, the regulatory body responsible for medicines and medical devices in the UK, perceive symptom checkers to be low-risk medical devices^29^. As such, these systems are only subject to self-certification prior to entering the open market. As highlighted by our results, there is a considerable clinical risk that can be associated with the use of these systems. Despite disclaimers, these systems clearly provide end-users with different diagnoses and behavioural recommendations. Symptom checkers, including testing and validation data and other premarket submissions, should be scrutinised and approved through regulatory body review, aided by independent experts; akin to the process that is required for Software as Medical Devices approval from the FDA^30^. Greater regulatory scrutiny must centre around clear reporting of diagnostic performance, the datasets used to train and validate the systems as well as the clinical context in which evaluations were undertaken. Viewing these systems as a higher class of medical devices would also allow mandate greater post-market surveillance scrutiny, which will supersede the current status quo of anecdotal reports through media sources.

#### For developers of symptom checkers

Robust clinical validation and testing is warranted to improve current software trustworthiness and reliability. The responsibility of this process lies with companies that develop such technologies; a process that is akin to how pharmaceutical companies are expected to provide data supporting the efficacy and safety of drugs to regulators.

The development of symptom checkers should ideally incorporate real-world patient data. Within their product life cycle, digital health technologies, particularly those which are AI-centred, typically go through a training phase, a validation phase, and a test phase. Within a training phase, a model (e.g., a symptom checker) learns a particular task (e.g., to identify or triage diagnoses) using a particular dataset (e.g., real or simulated examples of a particular clinical presentation). During the validation phase, further data is used to provide an unbiased evaluation of model accuracy, which allows developers to fine tune aspects of the model prior to committing to a final version. A test phase uses data that the model has not encountered before (e.g., new clinical data) in order to evaluate overall performance (e.g., diagnostic or triage accuracy). For a number of reasons, including financial and ease of access, some developers rely upon simulated vignettes or synthetic data in order to provide the data required for the aforementioned phases.

While vignettes or synthetic data with typical disease patterns and standardised presentations may be viewed as a viable and accessible means of training a symptom checker, this may not negate the need for testing using real-world cases^27^. Clinical vignettes are inherently limited in their utility to assess the diagnostic accuracy or triage safety of symptom checkers. Real-world evidence studies involving real patients have been recommended to examine the performance of symptom checkers against clinicians^27^. The importance of using real-world data within digital health technology development is also strongly supported by regulatory authorities, as evidenced by the introduction of the ‘Real World Evidence’ program by the FDA, which have also approved databases with real-world patient data to support this initiative^31–33^.

Obtaining a representative and balanced patient vignette set is especially pertinent for the training of symptom checkers and development of AI algorithms. The lack of quality training data and the presence of dataset imbalances will likely directly influence the diagnostic model and will negatively impact diagnostic and triage accuracy^34^. Building upon this, developers should use a wide and representative range of cases that encompass typical and atypical disease presentations; various demographic fractions (age, gender, and ethnicity) as well as medical comorbidities. These cases, which are collated in the form of datasets, should adhere to emerging guidance as to what constitutes transparent and complete reporting. Derived from the seminal ‘Datasheets for Datasets’ effort, the recent ‘Healthsheet’ initiative has set forth healthcare-specific standards around the reporting of dataset transparency and diversity^35,36^. This initiative has also been endorsed by the ongoing STANDING Together project, which will further serve to provide guidance around minimising racial and ethical health inequalities in AI datasets dataset composition^37^. These initiatives will prove crucial to regulators, commissioners, policymakers, and health data institutions who will have greater guidance as to how to assess whether the dataset is diverse and represents all segments of populations, including underserved groups. Conversely, creation of representative patient vignette sets will allow regulators to further validate symptom checkers during the approval process and ‘benchmark’ symptom checker performance. Providing developers access to a proportion of vignettes (i.e., a development dataset) will additionally improve transparency and understanding of cases that symptom checkers are expected to be able to process during the validation process.

#### For healthcare researchers

All the above recommendations offer healthcare researchers many avenues for further work. Within this, it is important that digital health studies that form the basis for symptom checkers are carried out with greater methodological rigour and transparency. First, vignettes used in audit studies examining symptom checker accuracy should be openly accessible. Within this systematic review, except for Semigran et al. and Hill et al., the vignettes that are inputted into symptom checkers are not published as supplementary data in the included studies. Thus, reproducibility and validation of audit study findings would be greatly aided by publishing the clinical vignettes used. Second, the majority of the included studies involved clinicians, not patients, inputting either clinical vignette details or reported symptoms from patient records into symptom checkers. However, these ‘clinician-vetted’ symptoms may differ from patients’ characterisation of their own physical symptoms and may therefore produce differing symptom checker results and limit generalisability. One potential avenue for further symptom checker validation could involve prospective data collection (i.e., from outpatient clinics and ED waiting areas) and comparison of results with clinician diagnoses. This approach would also be more representative of the role a symptom checker may play within the patient journey and a valuable method of determining patient attitudes and acceptability of symptom checkers. Third, to our knowledge, continued and persistent longitudinal assessment of symptom checker performance is not currently available in the literature. Several studies have suggested that symptom checkers performance may improve over time given increased exposure to data upon which an AI-centred model may iterate. This is especially pertinent as adaptive AI algorithms (where there is continuous learning from new data and subsequent algorithm modification) are incorporated into symptom checkers. Thus, these systems may have variable performance over time, which will require quantification with future longitudinal studies involving benchmarking clinical vignettes and pertinent population subgroup analyses. Fourth, while symptom checkers fulfil the need for telemedicine, further work should also evaluate whether symptom checkers truly are better than more traditional telephone triage lines. Previous work by Semigran et al. directly comparing physician and symptom checker (Human Dx) diagnostic accuracy noted significant outperformance by physicians^38^. Furthermore, comparison of symptom checker and search engine performance is needed. Search engines (e.g., Google, Bing, or Yahoo) are often a first port-of-call for self-diagnosis, though concerns regarding their use for this purpose have been raised^1,39,40^. More importantly, there is an unmet need for educating patients about using symptom checkers, especially about their limitations. While the variation in digital health literacy has previously been established, more effort is required to address and correct its socio-economic drivers.

Finally, one of the most important questions that have arisen from our review centres around what constitutes acceptable performance within this class of technologies, given that these technologies can be adopted across various clinical pathways and health systems, with differing degrees of clinical oversight. Given the variance in performance that we highlight across studies, it is imperative that the evidence produced by healthcare researchers and used by regulators, national policymakers and commissioners is transparent and complete, particularly around the reporting of reference standards and clinical context, which are key factors required for a health technology assessment. Although previous work has directly compared symptom checker and physician performance^38^, distinct evidence needs to be generated against specific clinical pathways and health systems in order to facilitate fair appraisals. These reporting and evidential tenets are upheld by guideline initiatives produced by The EQUATOR Network, such as CONSORT-AI and STARD-AI, which mandate the reporting of such key study characteristics for AI-centred clinical trial and diagnostic test accuracy studies, respectively^41,42^.

## Conclusions

In this systematic review, symptom checkers diagnostic and triage accuracy varied substantially and was generally low. Variation exists between different symptom checkers and the conditions being assessed; this raises safety and regulatory concerns. Given the increasing trend of telemedicine use and, even the endorsement of certain applications by the NHS, further work should seek to re-examine the regulation associated with these technologies as well as establish datasets to support their development and improve patient safety.

## Methods

### Eligibility criteria

This systematic review was conducted according to the Preferred Reporting Items for Systematic Reviews and Meta-Analyses (PRISMA) guidelines and was registered in the PROSPERO registry (ID: CRD42021271022)^43^. Prospective and retrospective vignette or audit studies were included. Studies that utilised an online or application-based service designed to input symptoms and biodata (i.e., age and gender) to generate diagnoses, health advice and direct patients to appropriate services were included. All study populations, including patients, patient cases or simulated vignettes (i.e., cases written by clinicians) were included. Studies were included regardless of the condition(s) being assessed or the symptom checker used. Included studies had to quantitatively evaluate the accuracy of the symptom checker service. Excluded articles included descriptive studies, abstracts, commentaries, and study protocols. Only articles written in the English language were included.

### Search

Following PRISMA recommendations, an electronic database search was conducted using MEDLINE and Web of Science to include articles up to 15 February 2021 (search strategy detailed in Supplementary text). Reference lists of the studies included in the review synthesis were examined for additional articles. Search results were then imported into Mendeley (RELX, UK) for duplicate removal and study selection. Screening of articles was performed independently by two investigators (W.W. and C.C.). Uncertainties were resolved through discussion with a third and fourth author (S.C. and V.S.).

### Data extraction and analysis

Key data were extracted and tabulated from the included studies, including details of study design, participants, interventions, symptom checkers used, comparators and reported study outcomes. The location of the study was determined based on the affiliation of the corresponding author. Data extraction was performed independently by two investigators (W.W. and C.C.). The primary outcomes of this systematic review were (1) the accuracy of symptom checkers for providing the correct diagnosis and (2) the accuracy of subsequent triage advice given (i.e., whether the acuity of the medical issue was correctly identified, and patients were signposted to appropriate services). The secondary outcome of assessing variation in recommendations within studies of consistent clinical input data can be calculated from these extracted outcomes. Due to the heterogeneity of the included studies’ design, methodology and reported outcomes, a meta-analysis was not performed. A narrative synthesis of the included studies and pre-specified outcomes was instead carried out. Study bias was assessed using the Quality Assessment of Diagnostic Accuracy Studies (QUADAS-2) tool^44^. The risk of bias was assessed across the four domains by two investigators (W.W. and S.C.), and any disagreements were discussed and resolved by a third author (V.S.). The risk of bias of each domain was categorised as low, unclear or high.

### Reporting summary

Further information on research design is available in the Nature Research Reporting Summary linked to this article.

## Supplementary information

Supplementary Material

Nature Reporting Summary

## Data Availability

The search strategy is available in the Supplementary material; any additional data are available upon request.
